# Deconstructing Multi-Scale Hybrid Fiber-Reinforced Coarse Aggregate UHPC: From Pore Structure Tailoring to Cross-Scale Toughening

**DOI:** 10.3390/ma19102171

**Published:** 2026-05-21

**Authors:** Jiyang Wang, Yalong Wang, Lingbo Wang, Yu Peng, Qi Zhang, Jingwen Shi, Xianmo Xu, Shuyu Lin

**Affiliations:** 1 Institute of Advanced Engineering Structures, Zhejiang University, Hangzhou 310058, China; 22312290@zju.edu.cn (Y.W.); 0013452@zju.edu.cn (Y.P.); 22360084@zju.edu.cn (J.S.); 22412186@zju.edu.cn (X.X.); 22412183@zju.edu.cn (S.L.); 2 Zhejiang Communications Construction Group Co., Ltd., Hangzhou 310007, China; wlingbo@zjjtgc.com; 3 Zhejiang Academy of Building Research & Design Co., Ltd., Hangzhou 310012, China; zhangqi_zjsjky@163.com

**Keywords:** ultra-high performance concrete, calcium carbonate whisker, coarse aggregate, steel fiber, cross-scale toughening

## Abstract

Ultra-high-performance concrete incorporating coarse aggregates (UHPC-CA) exhibits pronounced multi-scale heterogeneity and staged damage evolution. However, existing single-scale reinforcement strategies often fail to address the complete micro-to-macro fracture process, leaving a critical research gap in achieving full-stage crack control. To address this, this study introduces a novel cross-scale toughening strategy using hybrid steel fibers (SF) and calcium carbonate whiskers (CCW), and decouples the coupled influences of water-to-binder (W/B) ratio, coarse aggregate (CA), and multi-scale fibers via an orthogonal design. Mechanical properties, fiber dispersion, and pore structure are jointly characterized to establish structure–property relationships. An optimal composition (W/B = 0.32, CA = 18%, SF = 2%, CCW = 1%) is identified, achieving a balanced enhancement of strength and ductility. Results indicate that matrix densification is primarily controlled by W/B via pore refinement, while mechanical performance is governed by the interplay between fiber spatial uniformity and interfacial integrity; the roles of CA and CCW are clearly stress-state dependent. Furthermore, a novel cross-scale synergistic mechanism is revealed, in which micro-scale CCW regulates microcrack initiation and stabilizes the pre-peak response, whereas macro-scale SF dominates post-peak behavior through crack bridging and pull-out energy dissipation. This sequential activation enables a full-stage enhancement of tensile performance, shifting failure from brittle localization to pseudo-ductile multiple cracking. The findings provide a correlative framework for tailoring UHPC-CA through multi-scale hybrid reinforcement.

## 1. Introduction

Characterized by a highly densified matrix and the potent bridging action of short steel fibers (SFs), ultra-high-performance concrete (UHPC) exhibits superior mechanical capacity and durability [1,2], making it highly attractive for critical infrastructures such as long-span bridge girders, piers, and deck pavements [3,4]. However, the intrinsic formulation of UHPC—dictated by an exceptionally low water-to-binder ratio (W/B, typically 0.16–0.25 [5]) and massive binder consumption—inevitably triggers severe early-age autogenous shrinkage [6]. Such volumetric instability poses a profound threat to structural integrity. Incorporating coarse aggregates (CAs) has emerged as a viable mitigation strategy. The high elastic modulus of CA not only stiffens the matrix and offsets production costs [7] but also constructs a rigid internal skeleton that restrains shrinkage strains. Notably, substituting the fine matrix with CA can suppress 21-day autogenous shrinkage from 404.4 με to 251.3 με (approximately 40% reduction) [8], thereby broadening the scalability of UHPC for large-scale structural applications. Nevertheless, the synergy of CA and a low W/B ratio inherently compromises rheological properties and matrix homogeneity, intensifying the formation of microdefects within the aggregate–matrix interfacial transition zone (ITZ). Consequently, this exacerbates the difficulty of restraining crack propagation in UHPC-CA.

Concrete fracture is fundamentally a hierarchical evolutionary process: damage initiates as micro-defects form at the scale of gel and capillary pores, coalesces into meso-cracks within the mortar phase, and ultimately propagates as macro-cracks, leading to structural failure [9,10]. In conventional UHPC formulations, SF predominantly governs the arrest of macro-cracks, owing to its high tensile strength, elastic modulus, and aspect ratio [11,12,13]. Yet, when confronting the complex, multiscale damage mechanisms inherent in UHPC-CA, monoscale fiber reinforcement often fails to achieve a synergistic balance between strength and toughness [14]. Current efforts to optimize fiber hybridization mostly rely on blending identical fiber materials with varying geometries or dimensions [15]. For instance, while coupling straight and deformed (barbed/twisted) fibers yields only marginal improvements in flexural capacity [16], hybridizing SF of different lengths (e.g., 1.5% at 13 mm and 0.5% at 6 mm) optimizes compressive strength but severely degrades mixture workability [17]. Alternatively, blending heterogeneous fibers with contrasting moduli offers complementary benefits [18]. However, Deng et al. [19] reported that steel–polypropylene (PP) hybrid systems predominantly enhance compressive toughness with negligible or even detrimental effects on ultimate strength at higher dosages. Evidently, traditional hybridization protocols—constrained by limited dimensional scale—fail to capture micro-defect evolution throughout the material’s life cycle, often resulting in adverse hybridization effects.

To achieve effective cross-scale crack mitigation, multiscale hybrid reinforcement strategies have been extensively explored in various cementitious composites. In cement mortars, a ternary system comprising calcium carbonate (CaCO_3_) whiskers (CCWs), polyvinyl alcohol (PVA) fibers, and SF successfully established a sequential crack-arresting mechanism spanning micro-, meso-, and macro-levels [20,21,22]. Specifically, the incorporation of CCW not only boosted compressive strength by 37.1% but also facilitated the homogeneous dispersion of larger-scale fibers. Analogous synergistic toughening has been corroborated in ultra-lightweight foamed cement (yielding a >16% increase in fracture-initiation toughness) [23] and in recycled aggregate concrete (improving peak strain by 24.85%) [24]. More recently, this multiscale philosophy has permeated UHPC research. For example, the concurrent use of microscale steel wool and macroscale SF has been shown to increase flexural strength and toughness by over 50% [25,26]. Furthermore, the introduction of nano- and micro-fillers—such as nano-CaCO_3_ [27], nano-SiO_2_ [28], and graphene oxide [29]—demonstrates significant efficacy in refining pore networks and strengthening SF-matrix interfacial bonding. As a naturally occurring microfiber, CW is distinguished by its high aspect ratio, strength-to-weight ratio, and thermal stability [30]. Despite these advantages, current research on multi-scale fiber-reinforced UHPC has primarily focused on optimizing the interfacial bonding behavior between SF and the matrix. The specific synergistic effects of multi-scale fibers—particularly the multiscale synergy between micro-level CCW and macro-level SF within a coarse-aggregate UHPC matrix—remain largely uncharted territory. Crucially, a rigorous scientific delineation of how this hybrid network governs the evolution of pore structure, alongside the quantitative mechanisms linking “pore optimization, interface strengthening, and cross-scale toughening,” is conspicuously absent from the current literature.

To bridge these fundamental knowledge gaps, the primary objective of this study is to engineer a novel multiscale hybrid UHPC-CA by synergistically integrating micro-scale CCWs and macro-scale SFs, thereby achieving concurrent enhancements in mechanical robustness, tensile ductility, and volumetric stability. Employing an orthogonal experimental framework [31], this study systematically isolates and evaluates the coupled impacts of W/B ratio, CA volume, and fiber dosages (SF and CCW) on macroscopic mechanical performance, mesoscopic fiber dispersion, and microscopic pore architecture. Subsequent statistical correlation analyses elucidate the intrinsic mapping among pore characteristics, dispersion metrics, and bulk mechanical responses. Finally, by integrating comprehensive mechanical testing with multi-technique microstructural characterization (SEM and MIP), this work decodes the multiscale toughening mechanisms that drive the transition from micro-pore regulation to macroscopic fracture resistance. The insights derived herein establish a theoretical cornerstone for the customized design and structural application of multiscale fiber-reinforced, high-performance cementitious materials.

## 2. Materials and Methods

### 2.1. Raw Materials

The raw materials used to prepare the composite mixtures included a cementitious binder system, aggregates, micro- and macro-reinforcements, and chemical admixtures.

The binder system comprised P·II 52.5 Portland cement (Tongling Conch Cement Co., Ltd., Tongling, Anhui, China) exhibiting a specific surface area of 372.9 m^2^/kg, Grade I fly ash (Jianbi Power Plant Zhenjiang, Jiangsu, China), and silica fume (Aken International Trading Co., Ltd., Shanghai, China) with a SiO_2_ content exceeding 94%. For the aggregates, calcined medium-silica sand with a particle size distribution of 40–70 mesh (Tongliao Hongyuan Silica Sand Co., Ltd., Tongliao, Inner Mongolia, China) was utilized as the fine aggregate. Crushed basalt rock with a continuous grading of 5–10 mm was employed as the coarse aggregate. Table 1, Table 2, Table 3, Table 4 and Table 5 summarize the detailed chemical compositions of the cementitious materials and the mineralogical composition of the cement.

**Figure 1:**
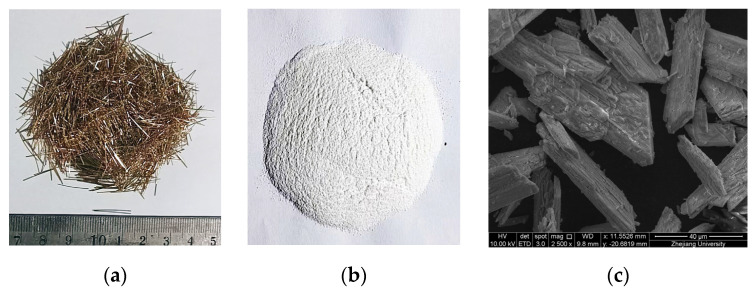
Representative morphology of reinforcing materials: (**a**) steel fibers; (**b**) CaCO_3_ whiskers; (**c**) microstructure of CaCO_3_ whiskers.

To enhance the mechanical performance of the matrix, two types of reinforcements were incorporated: copper-coated steel fibers (Zhejiang Boen Metal Products Co., Ltd., Huzhou, Zhejiang, China) and calcium carbonate whiskers (CCW) (Jinan Quanxin Chemical Co., Ltd., Jining, Shandong, China). The macro-scale physical properties of the steel fibers and the physical parameters of the CCW are detailed in Table 6 and Table 7, respectively, while their appearance and distinct micro-morphologies (e.g., the acicular structure of the whiskers) are illustrated in Figure 1.

Additionally, a dry-powder superplasticizer supplied by BASF was introduced to adjust the workability of the fresh mixtures, and standard tap water was used for all batches.

### 2.2. Mixture Proportion Design

#### 2.2.1. Particle Packing Optimization

The exceptional mechanical and durability characteristics of UHPC intrinsically rely on the physical densification of its multi-scale granular skeleton. To systematically tailor this spatial occupancy, the Modified Andreasen and Andersen (MAA) model [33,34] was adopted to guide the synergistic proportioning of solid precursors (i.e., cement, fly ash, silica fume, and sand). The ideal cumulative particle-size distribution (PSD) curve, *P*(*D*), serves as the theoretical target, and its expression is given by the following Equation (1):

(1) $$P\left(D\right)=\frac{D^{q}-D_{\min}^{q}}{D_{\max}^{q}-D_{\min}^{q}}$$

where *D* is the evaluated particle diameter (µm); *D*_max_ and *D*_min_ dictate the upper and lower boundary limits of the solid particle sizes within the dry mixture, respectively. The distribution modulus, *q*, fundamentally governs the packing geometry by regulating the ratio of coarse to fine fractions. Given the high dosage of ultra-fine supplementary cementitious materials in the formulated UHPC, *q* was empirically set to 0.23. This specific modulus is well documented to provide a favorable balance between the rheological workability and mechanical robustness of ultra-high-strength matrices [35].

In practice, the actual cumulative PSD of the multiphase granular system, *U*(*D*), is derived via the linear superposition of the volumetric fractions of individual components, expressed as Equation (2):

(2) $$U\left(D\right)=P_{Cement}\left(D\right)C_{Cement}+P_{FlyAsh}\left(D\right)C_{FlyAsh}+P_{SilicFume}\left(D\right)C_{SilicFume}+P_{Sand}\left(D\right)C_{Sand}$$

where *P_i_*(*D*) represents the discrete cumulative PSD of each raw material, accurately characterized using a Beckman Coulter LS13320 laser diffraction analyzer (Beckman Coulter, Inc., Brea, CA, USA), and *C_i_* denotes the volumetric proportion of the corresponding constituent. To navigate the trade-off between packing efficiency and material cost-effectiveness, an experimental matrix comprising 18 candidate mix proportions was formulated (Table 8). This matrix methodically explores six ternary binder configurations across three distinct binder-to-sand ratios.

**Figure 2:**
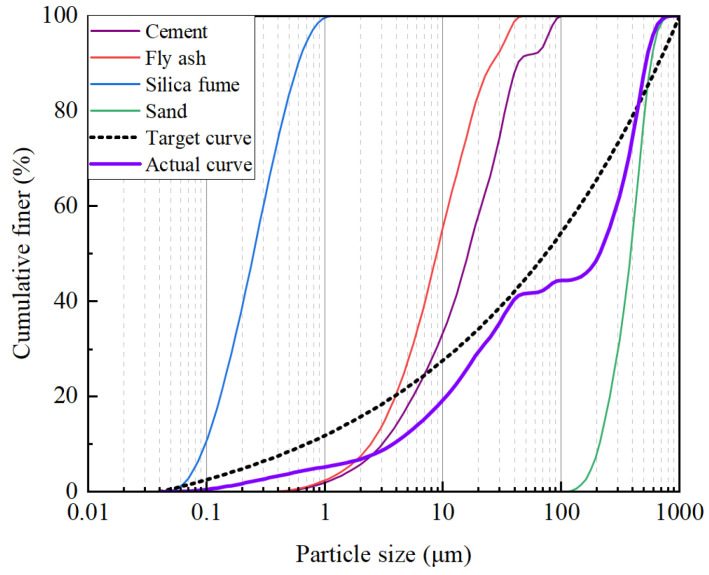
Comparison between the theoretical target curve and the optimized actual PSD curves.

To quantitatively bridge the gap between the theoretical optimum and the formulated blends, a Least Squares Method (LSM) algorithm was implemented. The objective function seeks to minimize the residual sum of squares (*RSS*) between the theoretical target *P*(*D*) and the actual blended curve *U*(*D*):

(3) $$RSS=\sum_{i=1}^{n}{\left[P\left(D\right)-U\left(D\right)\right]}^{2}\to \min$$

where *n* represents the number of discretized particle size intervals obtained from laser analysis. Minimizing the *RSS* enables the identification of the closest approximation to the ideal packing state. As illustrated in Figure 2, the optimized mixture—comprising 75% cement, 15% fly ash, and 10% silica fume with a binder-to-sand ratio of 80:20—exhibited the best agreement with the target PSD curve. This optimized gradation forms a highly compact granular skeleton, which is fundamental to enhancing the mechanical strength and durability characteristics of UHPC.

#### 2.2.2. Orthogonal Experimental Design

To establish a robust linkage between particle-scale packing, meso-scale aggregate skeleton, and fiber-mediated toughening, an orthogonal experimental design was employed to screen the dominant mixture parameters governing the multi-scale architecture of UHPC. Orthogonal experimental design enables decoupling of factor effects through a reduced yet statistically representative set of mixtures, thereby ensuring both experimental efficiency and interpretability [31].

In this study, four critical variables were selected, including the water-to-binder ratio (W/B), coarse aggregate content (CA), steel fiber dosage (SF), and calcium carbonate whisker content (CCW). These parameters respectively regulate matrix compactness, load-bearing skeleton formation, and crack-bridging capacity across multiple length scales. An L_16_(4^4^) orthogonal array was adopted to systematically evaluate their individual contributions and relative significance, with factor levels detailed in Table 9.

It is worth noting that CA was defined as a mass-based parameter (normalized to a reference mixture of 2500 kg), whereas SF and CCW were introduced as volumetric fractions to more accurately capture their reinforcing efficiency and spatial distribution within the matrix.

#### 2.2.3. Full Factorial Design

Building on the optimal parameter combination identified by orthogonal analysis, a full factorial design (as detailed in Table 10) was implemented to elucidate the coupling mechanisms among hybrid fibers at different scales. Fibers are essential for imparting tensile resistance and strain-hardening behavior to UHPC, particularly through multiscale crack bridging and energy-dissipation mechanisms [36]. To explicitly quantify the synergistic effects between macro-scale SFs and micro/nano-scale CCWs, a two-factor, multi-level full factorial matrix was constructed. As summarized in Table 10, nine mixtures were designed by varying the volumetric contents of SF and CCW.

This design enables not only the isolation of primary effects but also the identification of interaction effects between the two reinforcing phases. More importantly, it provides a quantitative basis for correlating fiber hybridization with crack evolution pathways and cross-scale toughening behavior, thereby offering mechanistic insight into the design of UHPC-CA.

### 2.3. Specimen Preparation and Testing Methods

#### 2.3.1. Specimen Preparation

The manufacturing protocol for the multi-scale UHPC-CA mixtures was strictly executed in accordance with the T/CBMF 37-2018 standard [37]. Initially, the granular skeleton, consisting of fine sand and coarse aggregate (CA), was dry-homogenized in a mixer for 30 s. Subsequently, the binder system—comprising cement, fly ash, silica fume, and calcium carbonate whiskers (CCW)—was introduced and dry-mixed for an additional 60 s to ensure uniform spatial distribution of the micro- and nanoscale precursors. Following the dry phase, approximately two-thirds of the pre-blended mixing water with the superplasticizer was added incrementally. The mixture was agitated for 3 min to activate the plasticizer and achieve initial flowability. The residual water was then incorporated, followed by another 3 min of mixing to further enhance paste homogeneity. Finally, the macro-scale steel fibers (SFs) were gradually dispersed into the matrix, with mixing sustained for 5 min to mitigate fiber agglomeration.

The freshly prepared UHPC-CA was cast into oiled molds and subjected to manual vibration to eliminate any entrapped macroscopic air voids. Immediately after casting, the exposed surfaces were sealed with polyethylene film to prevent plastic shrinkage induced by moisture evaporation. Specimens were demolded after 24 h of standard curing and subsequently submerged in a water bath until reaching the designated testing ages of 7 and 28 days.

#### 2.3.2. Mechanical Property Testing

**Figure 3:**
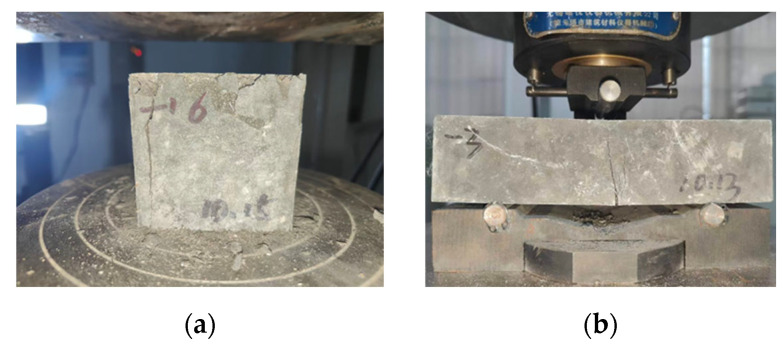
Configurations of specimens used for compressive and flexural tests: (**a**) compressive specimen; (**b**) flexural specimen.

Determinations of compressive and flexural strengths were performed following the GB/T 50081-2019 [38] and GB/T 31387-2025 [39] protocols, respectively. Compressive testing was conducted on 70.7 × 70.7 × 70.7 mm cubic specimens at a load-controlled rate of 0.6 MPa/s (three replicates per mixture). Flexural evaluations used 40 × 40 × 160 mm prismatic specimens loaded at 0.10 MPa/s; the testing configuration is schematically depicted in Figure 3.

**Figure 4:**
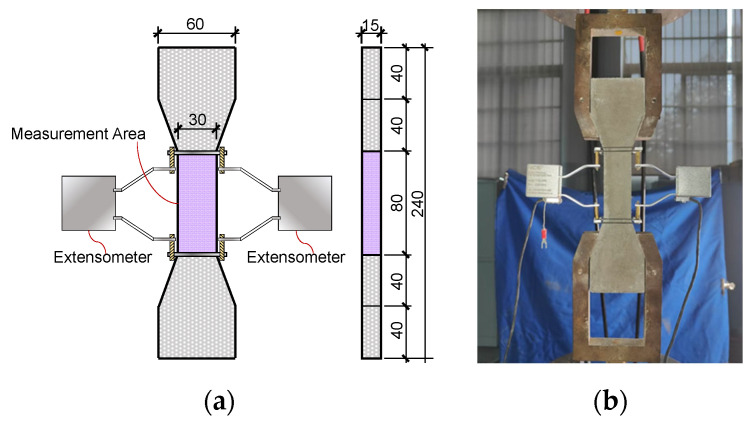
Geometry of tensile specimen and experimental setup: (**a**) specimen dimensions; (**b**) tensile test configuration.

To capture the uniaxial tensile response and strain-hardening behavior, dog-bone specimens (geometry shown in Figure 4a) were tested in accordance with JC/T 2461-2018 [40]. A displacement-controlled rate of 0.5 mm/min was applied. As illustrated in Figure 4b, a dual-extensometer setup was symmetrically attached to the central gauge length to continuously monitor tensile deformation during loading. For each mixture, compressive and flexural tests were performed in triplicate, while tensile tests were conducted using five replicate specimens.

#### 2.3.3. Fiber Dispersion Properties

The spatial architecture of the reinforcing phases fundamentally dictates the damage evolution and macroscopic ductility of UHPC [40]. A uniformly dispersed fiber network ensures optimal stress transfer and interfacial energy dissipation [41,42] while mitigating the local stress concentrations typically triggered by severe fiber agglomeration [43,44,45,46]. To elucidate the physical origin of the proposed cross-scale toughening mechanism, the three-dimensional (3D) mesoscopic distribution of SF within the tailored matrix was quantitatively evaluated.

**Figure 5:**
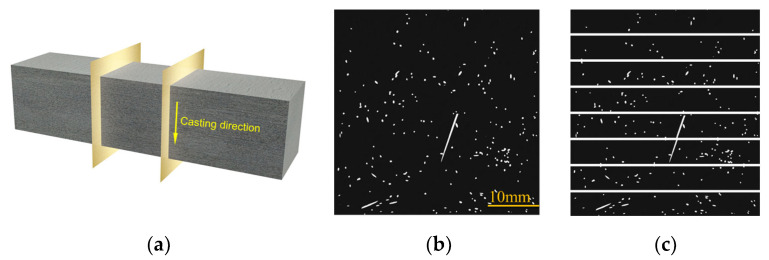
Quantitative evaluation of fiber dispersion: (**a**) extraction of cross-section; (**b**) binarized CT image; (**c**) segmented image for statistical analysis.

High-resolution X-ray computed tomography (X-CT, XTH 225/320 LC, Nikon Metrology UK Ltd., Tring, UK) was employed to non-destructively scan the core regions of the specimens. The acquired volumetric data were subsequently reconstructed and thresholded using VGStudio MAX software (version 3.1) to visualize the spatial topology of the SF network [47]. For quantitative analysis, virtual slices were extracted approximately 2 cm away from the primary fracture surface (Figure 5a). Each cross-section was digitally partitioned into eight identical subdivisions along the casting direction (Figure 5b,c). The fiber dispersion coefficient α was computed to characterize the distribution uniformity. Taking the average from two representative cross-sections, α is defined as in Equation (4) [40].

(4) $$\alpha =\exp\left(-\sqrt{\frac{\sum {\left(x_{i}/x_{avg}-1\right)}^{2}}{t}}\right)$$

where *x_i_* represents the localized fiber count within the *i*_-th_ subdivision, *x*_avg_ denotes the global average of fibers across all evaluated zones, and *t* is the total number of subdivisions (*t* = 8 in this study). The parameter α bounds between 0 and 1, with values approaching unity indicating a theoretically ideal, homogeneous dispersion state.

#### 2.3.4. Pore Structure Characterization

To correlate macroscopic mechanical performance with matrix compactness, representative fragments (approximately 2 g) were extracted from the cores of fractured compressive specimens. The internal hydration process was completely arrested by drying the samples at 60 °C for 14 days, eliminating evaporable water without inducing thermal microcracking. Subsequently, the multi-scale pore-size distribution and total porosity were precisely quantified using Mercury Intrusion Porosimetry (MIP; AutoPore IV 9510,Micromeritics Instrument Corporation, Norcross, GA, USA).

## 3. Results and Discussion

### 3.1. Mechanical Property Development

#### 3.1.1. Age-Dependent Strength Evolution

**Figure 6:**
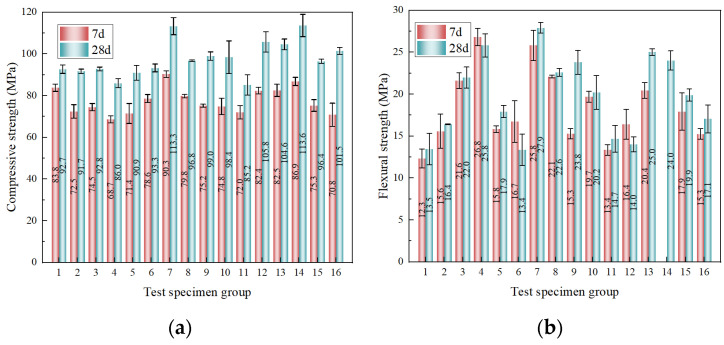
Age-dependent development of mechanical properties: (**a**) compressive strength; (**b**) flexural strength.

The compressive and flexural strengths at 7 d and 28 d are illustrated in Figure 6, with detailed numerical values and their standard deviations (SD) provided in Table 11. The ratio of 7 d to 28 d compressive strength (Figure 6a) ranges from 0.698 to 0.904, while that of flexural strength (Figure 6b) varies between 0.642 and 1.252. These results indicate that the developed UHPC-CA exhibits pronounced early-age strength development, accompanied by a relatively limited gain in strength at later ages. This behavior can be attributed to the synergistic effects of dense particle packing and early hydration reactions. At early ages, the incorporation of mineral admixtures (e.g., silica fume) promotes rapid formation of a compact matrix, while the presence of fibers and coarse aggregates contributes to an effective load-transfer skeleton. At later ages, strength enhancement is mainly governed by secondary hydration and pozzolanic reactions, which further densify the matrix and refine the interfacial transition zones (ITZs) between matrix, fibers, and aggregates.

Notably, for certain mixtures, the flexural strength at 7 d approaches or even exceeds that at 28 d. This phenomenon is primarily associated with fiber-rich or aggregate-rich systems, where initial heterogeneities (e.g., fiber clustering or local packing defects) are not fully mitigated during subsequent hydration. As a result, the potential for further strength enhancement becomes limited, particularly for flexural performance, which is highly sensitive to mesoscale defects.

#### 3.1.2. Orthogonal Range Analysis and Multi-Scale Mechanisms

**Figure 7:**
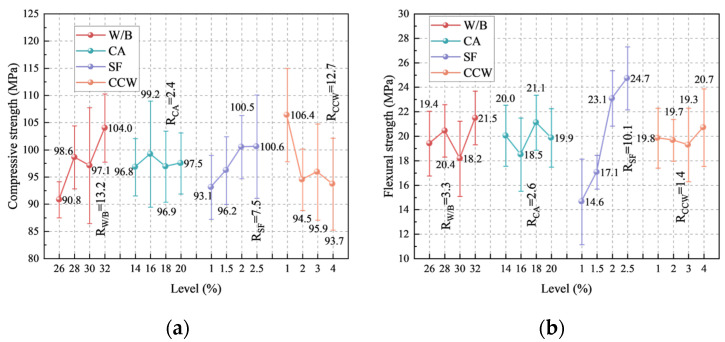
Orthogonal range analysis indicating the sensitivity of mechanical responses to multiscale variables: (**a**) compressive strength; (**b**) flexural strength.

To decouple the sensitivity of mechanical properties to the multiscale variables, a range analysis (R) was conducted on the 28 d strengths (Figure 7, Table 12). The hierarchical significance of the factors altering compressive strength is sequentially W/B (R = 13.2) > CW (R = 12.7) > SF (R = 2.4) > CA (R = 2.4). Conversely, the flexural strength is governed by a completely different hierarchy: SF (R = 10.1) > W/B (R = 3.3) > CA (R = 2.6) > CW (R = 1.4). This discrepancy clearly underscores that compressive resistance is predominantly dictated by matrix compactness (controlled by W/B and micro-fillers), whereas flexural toughness is fundamentally governed by macro-crack bridging capacity (dictated by SF).

Based on the orthogonal sensitivity and cross-scale reinforcing mechanisms, the optimal mixture parameters were determined as follows:

(1) W/B ratio (Optimal: 0.32)

Both compressive and flexural strengths peaked at a W/B of 0.32. This value significantly deviates from the ultra-low W/B (typically ~0.17) commonly reported for conventional UHPC [48]. This divergence is primarily attributable to the restricted superplasticizer dosage (1.0% of the cementitious binder by weight) used in this study. Under restricted chemical dispersion, a drastically reduced W/B ratio triggers excessive plastic viscosity and yield stress, leading to a substantial volume of entrapped air that cannot be expelled by vibration [49]. Consequently, a W/B of 0.32 provides the optimal rheological-hydrational balance, mitigating porosity while ensuring sufficient binder reaction.

(2) CW content (Optimal: 1.0 vol.%)

Calcium carbonate whiskers (CCW) exerted a profound influence on compressive strength but had a negligible effect on flexural capacity. The optimal dosage was identified as 1%. At the microscale, CCW acts not merely as a physical microfiller to refine capillary pores [9] but also as a microcrack arrestor. More importantly, as indicated by previous studies [50], the locally dissolved CO_3_^2−^ from the CCW surface can react with aluminate phases in cement to precipitate mono-carboaluminates. This synergistic physical-chemical nucleation accelerates hydration and further densifies the ITZ. However, exceeding 1% causes severe whisker agglomeration, leading to weak interfaces that adversely affect the compressive matrix.

(3) SF dosage (Optimal: 2.0 vol.%):

As expected, SF completely dominated the flexural performance. As a macro-scale reinforcing phase, SF possesses exceptional tensile capacity, effectively bridging macroscopic propagating cracks and dissipating fracture energy, thus imparting pseudo-ductility to the UHPC [17,51]. Although 2.5% SF yielded the absolute maximum strength, the marginal performance gain from 2.0% to 2.5% was notably smaller than the increase from 1.5% to 2.0%. Balancing mechanical efficiency, economic feasibility, and the rheological constraints discussed in Section 2.2, 2.0 vol.% was selected as the optimal dosage.

(4) CA content (Optimal: 18 wt.%):

The inclusion of CA introduces a meso-scale rigid skeleton within the UHPC, characterized by a high elastic modulus [49]. Range analysis revealed that an 18% CA addition maximizes flexural strength. Beyond this critical threshold, the limited paste volume fails to adequately encapsulate the coarse aggregates [52], leading to overlapping ITZs and skeleton instability, which consequently deteriorates the overall load-bearing capacity.

The analysis of variance (ANOVA) results for the mechanical properties are summarized in Table 13. In this table, SS denotes the sum of squares, which represents each factor’s contribution to the total variation. DOF represents the degrees of freedom, and MS denotes the mean square, which is calculated by dividing SS by the corresponding DOF. The F-value is obtained by comparing the MS of each factor with the error term and is used to evaluate the statistical significance of the factor effect. Fa (3,3) represents the critical F-value at the corresponding significance level and degrees of freedom. A factor is considered statistically significant when its F-value exceeds the corresponding critical value. The significance symbols * and ** indicate significant and highly significant effects, respectively.

As shown in Table 13, the effect of SF on flexural strength reaches a statistically significant level, whereas the effects of the other factors on flexural strength, as well as the effects of all investigated factors on compressive strength, are not statistically significant within the selected factor ranges. This result may be attributed to the relatively narrow range of variation among the factor levels used in the L16 orthogonal design. Nevertheless, the ANOVA results still provide useful quantitative support for the range analysis. Specifically, W/B and CCW show stronger influence on compressive strength, which is mainly associated with matrix densification and pore refinement, whereas SF exhibits the most pronounced effect on flexural strength due to its macro-scale crack-bridging capacity. These findings further confirm that compressive performance is primarily governed by matrix compactness, while flexural performance is more strongly dependent on the crack-bridging effect of macro-scale fibers.

### 3.2. Fiber Dispersion Characteristics and Cross-Scale Synergistic Mechanisms

**Figure 8:**
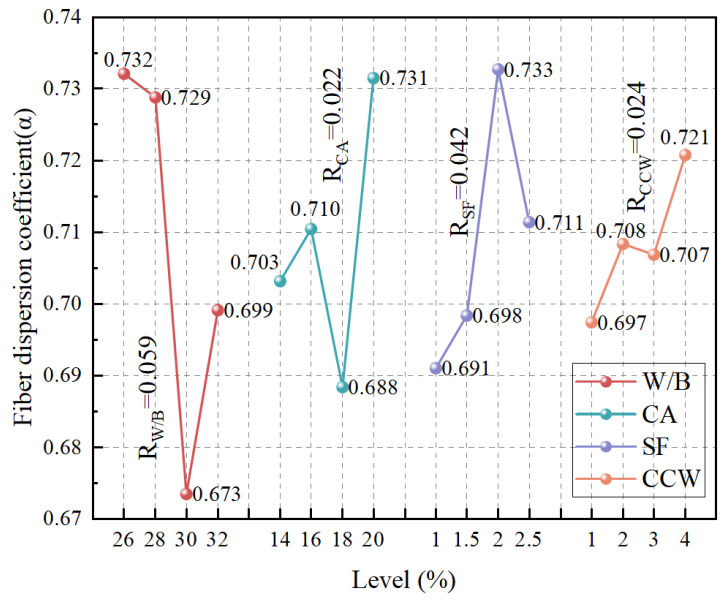
Range analysis of the fiber dispersion coefficient (α), evaluating the sensitivity to matrix composition and multiscale fiber dosages.

**Figure 9:**
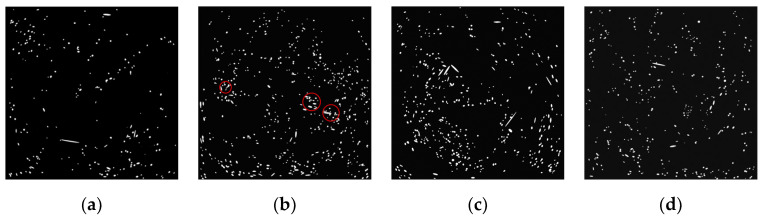
Representative binarized CT cross-sections revealing the mechanisms governing spatial fiber distribution: (**a**) fiber-depleted zones at insufficient dosage; (**b**) agglomeration (red circles) induced by high dosage; (**c**) gravity-driven segregation caused by an elevated W/B ratio; (**d**) optimal homogeneous dispersion architecture.

Figure 8 and Table 14 present the range analysis of the fiber dispersion coefficient (α), while Figure 9 illustrates the corresponding cross-sectional distributions reconstructed via X-ray CT. The sensitivity analysis reveals that the factors governing SF dispersion follow the sequence of water-to-binder (W/B) ratio > SF dosage > CW content > CA content.

The W/B ratio is the dominant factor, dictating the rheological environment of the fresh matrix. An elevated W/B ratio fundamentally reduces the yield stress and plastic viscosity of the suspending medium, thereby failing to counteract the gravitational sedimentation of the dense steel fibers. As corroborated by the CT slices, increasing the W/B from 0.28 (Figure 9b) to 0.30 (Figure 9c) induces severe vertical segregation, transitioning from a relatively uniform state to a pronounced heterogeneous distribution in which fibers accumulate heavily at the bottom of the casting.

The incorporation of coarse aggregates (CA) induces a parabolic effect on dispersion. Initially, the rigid granular skeleton formed by CA provides spatial confinement that physically obstructs the downward migration of SF, aligning with the barrier effect reported in [7]. However, exceeding the optimal CA threshold disrupts the local flow field during mixing, mechanically hindering the homogenization of fibers within the cementitious paste.

Regarding the SF dosage, a clear threshold behavior is observed. At a low dosage (e.g., 1%, Figure 9a), the low spatial collision probability results in localized fiber-depleted zones. Conversely, an excessive dosage (e.g., 2.5%, Figure 9b) dramatically increases inter-fiber friction and mechanical entanglement, ultimately resulting in detrimental agglomeration (highlighted by red circles). An optimal dosage of 2% (Figure 9d) strikes a balance between spatial reinforcement density and mixing efficiency, achieving a highly homogeneous architecture (α = 0.792).

Most importantly, the addition of micro-scale CCW steadily enhances the dispersion of macro-scale SF, highlighting a unique cross-scale structural synergy. This enhancement originates from a dual mechanism: geometrically, the fine CCW particles optimize the solid packing and increase the tortuosity of the localized settling paths for SF [9]; mechanically, the randomly oriented micro-whiskers form a robust suspension network around the macro-fibers. This localized interlocking at the micro-scale effectively suspends the SF and mitigates gravity-induced settlement [21], demonstrating that multi-scale hybridization not only contributes to hardened mechanics but also fundamentally improves the fresh-state structural stability.

### 3.3. Microstructural Characterization: Pore Structure Evolution

#### 3.3.1. Total Porosity and Matrix Densification

**Figure 10:**
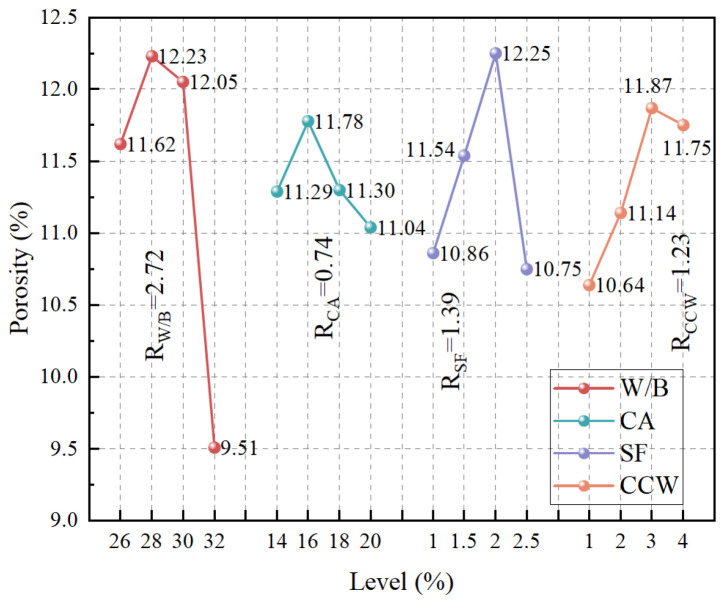
Orthogonal range analysis of total porosity.

Total porosity serves as a critical macroscopic indicator of matrix compactness and defect accumulation. Based on the orthogonal range analysis (Figure 10), the sensitivity hierarchy dictating the porosity of the UHPC-CA matrix is W/B (R = 2.72) > SF (R = 1.39) > CCW (R = 1.23) > CA (R = 0.74).

Consistent with the mechanical strength findings, elevating the W/B ratio (within the tested range of 0.32) paradoxically reduces total porosity. This further corroborates the rheological constraints discussed earlier: under a restricted superplasticizer dosage, an ultra-low W/B ratio yields a highly viscous paste that traps a substantial volume of macroscopic air voids during mixing. Increasing the W/B ratio effectively relieves the yield stress, facilitating the expulsion of entrapped air while simultaneously providing sufficient moisture for a higher degree of secondary hydration of silica fume.

Conversely, the incorporation of SF, CCW, and CA generally induces an upward trend in porosity, albeit with minor fluctuations. This phenomenon is fundamentally driven by the “wall effect” and the proliferation of interfacial transition zones (ITZs). ITZs intrinsically possess higher porosity and lower packing density than the bulk paste. Furthermore, although an optimal dosage of CCW exhibits well-documented micro-filling and hydration-promoting effects [9,50], excessive CCW or SF content increases the surface area requirement and induces severe local agglomeration or fiber entanglement, thereby creating additional voids and hindering matrix consolidation.

#### 3.3.2. Multiscale Pore Size Distribution and Refinement Mechanisms

Beyond total porosity, the topological distribution of pore sizes critically governs the macroscopic fracture mechanics of UHPC-CA. Following established micromechanical classifications [53], the void network is categorized into gel pores (<10 nm), small capillaries (10–100 nm), large capillaries (100–1000 nm), and macropores (>1000 nm). Pores smaller than 100 nm are generally designated as “harmless pores”, whereas those exceeding 100 nm act as local stress concentrators and are deemed “harmful pores.”

**Figure 11:**
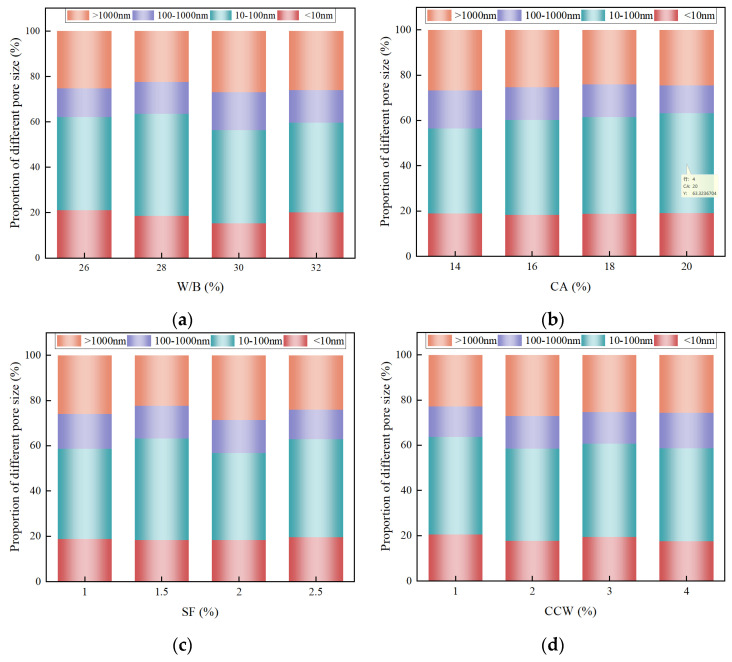
Evolution of pore size distribution decoupled by multiscale variables: (**a**) W/B ratio; (**b**) CA content; (**c**) SF content; (**d**) CCW content.

Figure 11 reveals a distinct, decoupled scaling effect of the different mixture variables on the pore size distribution. Notably, variations in the W/B ratio and CCW dosage synchronously alter the volume fractions of both gel pores and small capillaries. This is mechanistically sound: the W/B ratio dictates the intrinsic stoichiometry of the cementitious system, thereby directly governing the volume of precipitated C–S–H gel. Concurrently, the chemically active CCW not only acts as a physical nucleation site but also undergoes surface dissolution, reacting with aluminate phases to form mono-carboaluminates [9,50]. This synergistic physico-chemical reaction intricately refines the nanoscale gel pore network.

In contrast, adjusting the meso-to-macroscale inclusions (CA and SF) predominantly perturbs the fraction of small and large capillary pores, leaving the nanoscale gel pore fraction virtually invariant. This observation provides compelling evidence that inert coarse aggregates and steel fibers do not alter the fundamental thermodynamics of hydration. Instead, their impact is physically confined to modifying the spatial packing density, altering ITZ thickness, and inducing structural capillary voids due to geometric mismatch.

Consequently, a synergistic multiscale optimization strategy can be derived: utilizing an appropriate W/B ratio and active micro-scale CCW to chemically densify the intrinsic nanoscale gel network, while simultaneously optimizing the macro-scale CA and SF fractions to minimize meso-scale ITZ defects. This cross-scale structural tailoring maximizes the proportion of harmless pores, ultimately yielding an ultra-dense matrix with superior load-bearing capacity.

### 3.4. Structure–Property Relationships: Coupling Effects of Porosity and Fiber Dispersion

**Figure 12:**
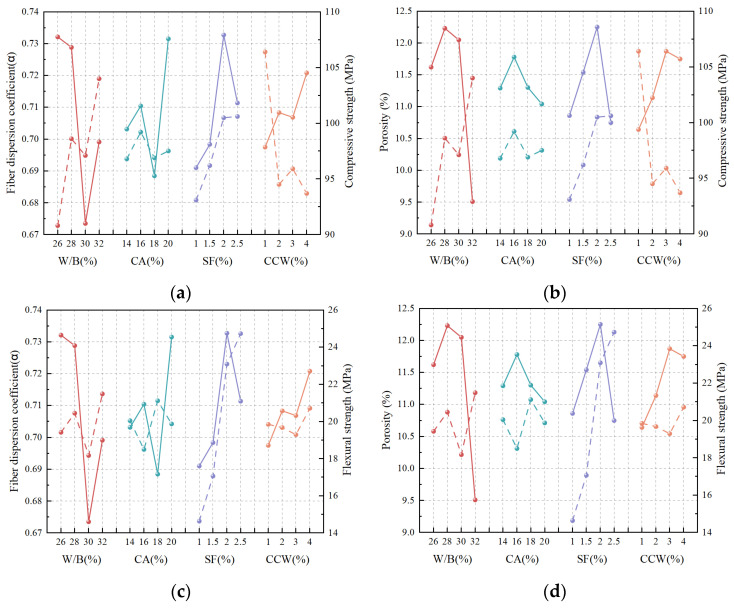
Correlation analysis revealing the multi-scale structure-property relationships: (**a**,**b**) competing effects of fiber dispersion and porosity on cube compressive strength; (**c**,**d**) synergistic effects of fiber dispersion and porosity on flexural strength. Solid and dashed lines represent the relationships for the corresponding metrics as indicated by the left and right y-axes, respectively, while different colors denote different x-axis factors.

To fundamentally elucidate the reinforcing mechanisms of the multi-scale hybrid system, Figure 12 correlates the micro/meso-structural parameters (porosity and fiber dispersion coefficient, α) with the macroscopic mechanical responses under varying mixture designs.

Compressive failure in UHPC-CA is essentially driven by the propagation and coalescence of internal micro-defects under shear-compressive stress states. Consequently, compressive strength is highly sensitive to matrix compactness. As illustrated in Figure 12a,b, the optimization of the W/B ratio (0.32) and the addition of CCW (1%) enhance the compressive capacity primarily through a substantial reduction in porosity. In this regime, the micro-scale CCW acts predominantly as an active filler, refining capillary voids and densifying the matrix rather than functioning as a spatial dispersant. Conversely, the incorporation of CA (16%) and SF (2%) improves the compressive strength mainly by maximizing the fiber dispersion coefficient (α). A highly uniform fiber architecture establishes an effective three-dimensional load-transfer skeleton, mitigating localized stress concentrations and restraining the matrix’s lateral expansion under compression.

In sharp contrast to the compressive response, flexural strength is intrinsically dictated by the material’s post-cracking tensile capacity, making it fundamentally dependent on the spatial distribution of the bridging phases. Figure 12c,d reveal that the flexural enhancements induced by higher SF (2%) and CCW (4%) dosages are strongly coupled with improved fiber dispersion. A homogeneous multi-scale fiber network eliminates weak planes and guarantees multi-crack bridging, thereby maximizing fracture energy dissipation. Meanwhile, the W/B ratio and CA content contribute to flexural performance largely through pore refinement. This matrix densification physically strengthens the fiber–matrix interfacial transition zone (ITZ), which critically enhances the pull-out frictional resistance of the well-dispersed steel fibers.

Synthesizing the above observations, a distinct mechanistic transition is identified within the hybrid system. The W/B ratio universally governs mechanical performance through microstructural pore refinement, whereas SF dominates load-bearing capacity by establishing robust mesoscale crack-bridging networks. Interestingly, the functional roles of CA and CCW exhibit strong stress-state dependence—dynamically shifting between physical packing (matrix densification) and structural scaffolding (promoting spatial fiber distribution) depending on whether the system is under compression or flexure.

### 3.5. Direct Tensile Response and Multiscale Fracture Mechanisms

#### 3.5.1. Macroscopic Fracture Morphologies and Crack-Bridging Behavior

**Figure 13:**
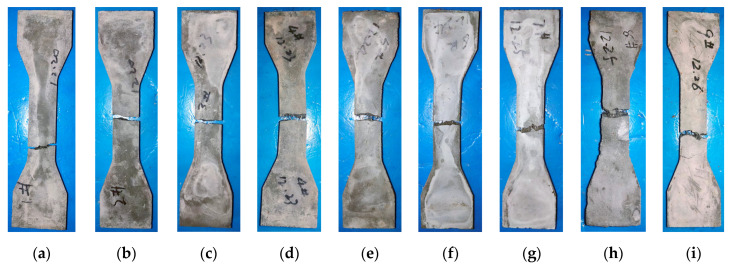
Macroscopic failure modes of direct tensile specimens under varying multiscale fiber hybridizations: (**a**) SF0CCW0; (**b**) SF0CCW1; (**c**) SF0CCW2; (**d**) SF1CCW0; (**e**) SF1CCW1; (**f**) SF1CCW2; (**g**) SF2CCW0; (**h**) SF2CCW1; (**i**) SF2CCW2.

**Figure 14:**
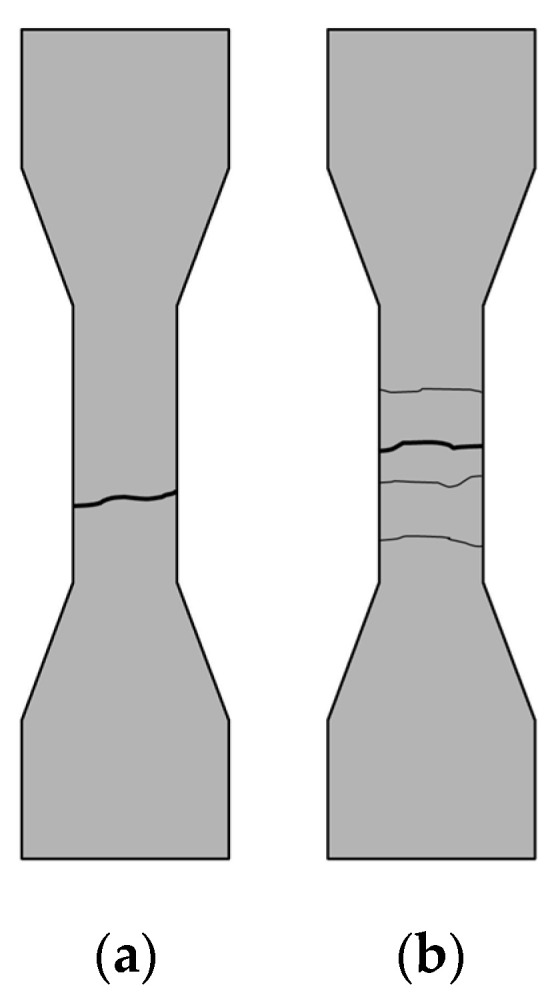
Comparison of macroscopic fracture morphologies demonstrating the transition of failure modes: (**a**) typical brittle failure with a single localized crack in the unreinforced matrix (SF0CCW0); (**b**) pseudo-ductile failure characterized by multiple-cracking behavior driven by optimal multi-scale fiber bridging (SF2CCW1).

The macroscopic failure morphologies of the tensile specimens are comparatively illustrated in Figure 13 and Figure 14. For the unreinforced matrix and mixtures solely incorporating micro-scale CCW (Figure 13a–c), the specimens exhibited typical brittle fracture, characterized by a single, highly localized main crack (as schematically shown in Figure 14a). This indicates that the micro-CCWs alone are insufficient to alter the inherent brittleness of the UHPC-CA matrix.

Conversely, the incorporation of macro-scale SFs (Figure 13d,g) fundamentally transitioned the fracture kinematics from brittle to pseudo-ductile. A substantial number of steel fibers effectively bridged the fracture surfaces, restraining sudden structural collapse. Notably, in the optimized hybrid system (SF and CCW blended, e.g., Figure 13e,h), although the ultimate failure was still governed by macro-crack localization, a distinct multiple-cracking characteristic emerged prior to failure (Figure 14b). This suggests that while SF spatially governs the global failure mode, the synergistic presence of CCW alters the pre-peak crack propagation behavior.

#### 3.5.2. Tensile Capacity and Cross-Scale Synergistic Effects

**Figure 15:**
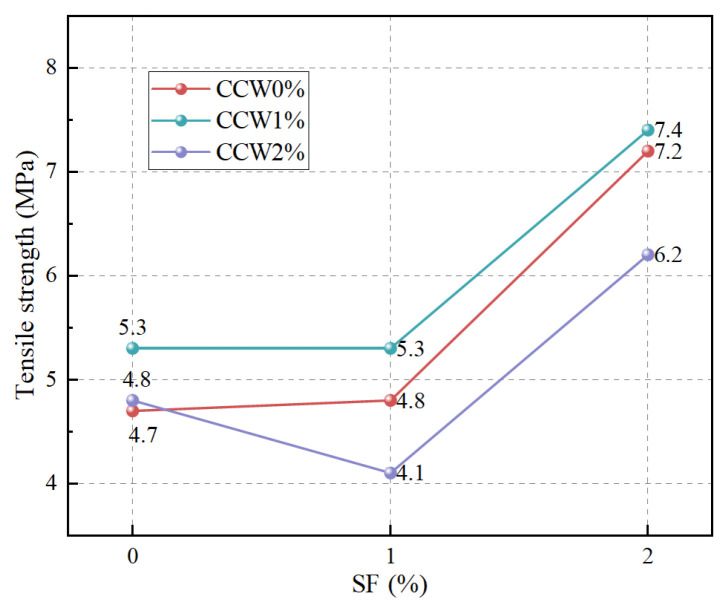
Tensile strength as a function of SF and CCW contents, illustrating the cross-scale hybrid effect.

The evolution of ultimate tensile strength under varying multiscale fiber dosages is presented in Figure 15, with Table 15 providing the specific tensile strength and standard deviation (SD) for each group of specimens. The mono-incorporation of CCW exerts a marginal influence on the tensile capacity, whereas SF dictates the primary tensile strength enhancement. Crucially, a pronounced positive hybridization effect is observed at the optimal mix ratio (SF 2% + CCW 1%), where the tensile strength reaches its maximum (7.4 MPa), which aligns with the orthogonal optimization findings in Section 3.1.2.

From a micromechanical perspective, this non-linear enhancement stems from a cross-scale structural synergy. Micro-CCWs function as intrinsic reinforcement, bridging internal micro-defects and homogenizing the stress field near the matrix–aggregate interfaces. Meanwhile, macro-SFs provide extrinsic toughening by bearing the principal tensile stress across developing macro-cracks. However, deviating from this optimal ratio (e.g., excessive CCW) induces whisker agglomeration, leading to localized interfacial flaws and compromising the overall load-bearing capacity. This threshold-dependent synergistic pattern strongly resonates with the hybridization mechanisms reported in PE fiber–calcium sulfate whisker (CSW) composite systems [54], further underscoring the necessity of geometric and volumetric matching in multiscale composites.

#### 3.5.3. Constitutive Response and Multi-Stage Toughening Conceptualization

**Figure 16:**
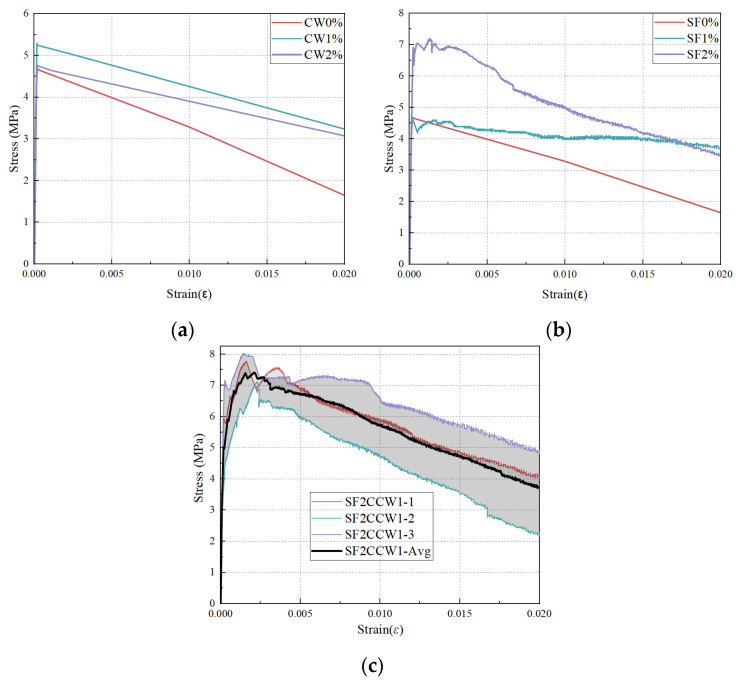
Direct tensile stress–strain curves under varying fiber configurations: (**a**) variable CCW contents without SF; (**b**) variable SF contents without CCW; (**c**) optimized SF2CCW1 system exhibiting robust pseudo strain-hardening.

**Figure 17:**
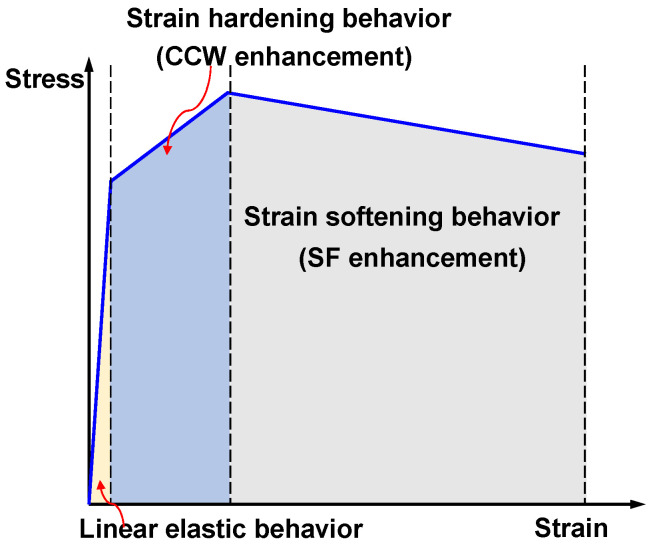
Conceptual diagram of the cross-scale synergistic toughening mechanism during the direct tensile fracture process.

To deeply decipher the synergistic toughening mechanisms, the direct tensile stress–strain (*σ*–*ε*) curves are analyzed (Figure 16) and conceptualized into three governing stages: linear elastic, pseudo strain-hardening (PSH), and strain-softening behaviors (Figure 17).

As depicted in Figure 16a, mixtures without SF lack post-cracking load-bearing capacity, exhibiting an abrupt stress drop post-peak. When solely reinforced with SF (Figure 16b), the specimens demonstrate a prolonged strain-softening region but limited PSH capacity. For instance, the stress increments from initial cracking to peak strength for SF 1% and SF 2% are merely −4.2% and 4.3%, respectively, indicating that mono-SF reinforcement is inefficient at delaying crack localization.

Remarkably, the optimized hybrid mixture (SF2CCW1, Figure 16c) completely reshapes the constitutive response, unlocking a robust PSH stage. The initial cracking strengths range from 5.9 to 7.2 MPa, followed by a substantial continuous stress increment up to 30%, culminating in peak strengths between 7.1 and 8.0 MPa.

This profound enhancement implies that the multiscale fibers exhibit spatio-temporal complementarity during the fracture process. Chronologically, the micro-scale CCW is activated during the pre-peak stage (PSH region), where it suppresses microcrack widening, raises the matrix’s initial cracking threshold, and enables the matrix to withstand higher strains before macrocrack localization. Spatially, once macro-cracks fully develop and the matrix softens (post-peak region), the high-strength macro-SF takes over, dissipating massive fracture energy through continuous frictional pull-out. This sequential activation—CCW strengthening the hardening phase and SF dominating the softening phase—maximizes the phase advantages of both materials, yielding an exceptionally tough UHPC-CA composite.

## 4. Conclusions

A multiscale hybrid UHPC-CA material incorporating macro-scale SFs and micro-scale CCWs was systematically developed. By linking matrix rheology, fiber dispersion, pore topology, and mechanical response, a unified cross-scale reinforcement framework is established that addresses the existing knowledge gap on multiscale synergy, facilitates the mix design of multi-scale UHPC-CA, and promotes the advanced application of multi-scale fibers in high-performance engineering structures. The main conclusions are as follows:

(1) Stress-state-dependent optimization: Compressive strength is governed by matrix densification (W/B and CCW), whereas flexural performance is controlled by crack-bridging capacity (SF and CA). An optimal balance is achieved at W/B = 0.32, CA = 18%, SF = 2%, and CCW = 1%.
(2) Rheology-controlled fiber dispersion: Fiber distribution is primarily dictated by matrix rheology rather than dosage alone. Increasing W/B lowers yield stress and induces gravity-driven SF segregation, while CA and CCW provide spatial confinement. A critical SF content (~2%) is required to avoid fiber depletion and agglomeration.
(3) Decoupled pore refinement mechanism: Pore evolution follows a physico-chemical decoupling. W/B and CCW regulate gel pores via hydration and nucleation, whereas CA and SF mainly affect capillary pores and ITZs through geometric packing, without altering the intrinsic gel network.
(4) Synergistic toughening: Under tension, CCW enhances the pre-peak regime by suppressing microcrack coalescence and promoting pseudo strain-hardening, while SF governs post-peak energy dissipation via crack bridging and pull-out. Their synergy (2% SF + 1% CCW) enables a transition from brittle fracture to stable multiple cracking.
(5) Future Perspectives: Future studies will continue to develop physics-informed constitutive models and employ advanced microstructural characterization (e.g., SEM-EDS, XRD, TGA) to deepen our understanding of chemical hydration kinetics and fiber-matrix interactions. From an engineering standpoint, future work will also specifically address production scalability, conduct comprehensive cost and environmental impact assessments of CCW utilization, and verify the composite’s durability and structural performance under realistic exposure conditions relevant to marine infrastructure. This will ensure its promising potential for extensive application.

## Figures and Tables

**Table 1:** Chemical composition and content of cement (%) [32].

Insoluble Matter	Loss on Ignition	MgO	SO_3_	Chloride Ion	Limestone	Natural Gypsum
0.95	2.13	1.46	2.59	0.019	2.16	6.54

**Table 2:** Mineralogical composition of the cement (%) [32].

C3S	C2S	C4AF	C3A	Calcite	Gypsum	Bassanite	Anhydrite	Free CaO	Free MgO
59.4	13.8	9.4	6.5	4.8	2.6	1.6	0.7	0.4	0.8

**Table 3:** Chemical composition and content of fly ash (%).

Moisture Content	Loss on Ignition	Water Requirement Ratio	SO_3_	Free CaO	Total Mass Fraction of SiO_2_, Al_2_O_3_, and Fe_2_O_3_	28 d Activity Index
0.02	1.80	93	0.7	0.05	77	79

**Table 4:** Chemical components and content of fine sand (%).

SiO_2_	Fe_2_O_3_	Al_2_O_3_	TiO_2_	CaO	MgO	Na_2_O	K_2_O
93.0	0.31	4.63	0.057	0.23	0.12	1.38	2.18

**Table 5:** Chemical components and content of micro-silica powder (%).

SiO_2_	Moisture Content	Loss on Ignition
94.0	0.4	0.5

**Table 6:** Physical property parameters of steel fiber [32].

Tensile Strength (MPa)	Length (mm)	Equivalent Diameter (mm)	Length-to-Diameter Ratio
2850	13.0	0.22	59

**Table 7:** Physical property parameters of calcium carbonate whiskers.

Purity (%)	Moisture Content (%)	Equivalent Diameter (µm)	Length-to-Diameter Ratio	pH	Heat Resistance (°C)
98	0.38	8	21	6.5	980

**Table 8:** Experimental matrix of raw material combinations for packing optimization.

Binding Materials			Binder-to-Sand Ratio (%)
Cement (%)	Fly Ash (%)	Silica Fume (%)	
80	15	5	70/75/80
80	12.5	7.5	70/75/80
80	10	10	70/75/80
75	20	5	70/75/80
75	17.5	7.5	70/75/80
75	15	10	70/75/80

**Table 9:** Factors and corresponding levels in the orthogonal experimental design.

W/B	CA (%)	SF (%)	CCW (%)
0.26	14	1	1
0.28	16	1.5	2
0.30	18	2	3
0.32	20	2.5	4

**Table 10:** Experimental matrix of the full factorial design—research program.

**Specimen Groups**	**SF (%)**	**CCW (%)**
SF0CCW0	0	0
SF0CCW1	0	1
SF0CCW2	0	2
SF1CCW0	1	0
SF1CCW1	1	1
SF1CCW2	1	2
SF2CCW0	2	0
SF2CCW1	2	1
SF2CCW2	2	2

Note: Compressive and flexural strength tests were performed using 3 specimens per group, whereas tensile tests utilized 5 specimens per group.

**Table 11:** Strength and SD results (MPa).

Specimen Groups	7 d Compressive Strength (SD)	28 d Compressive Strength (SD)	7 d Flexural Strength (SD)	28 d Flexural Strength (SD)
1	83.8 (1.75)	92.7 (2.06)	12.3 (1.13)	13.5 (1.86)
2	72.5 (3.23)	91.7 (1.07)	15.6 (2.05)	16.4 (0.05)
3	74.5 (1.70)	92.8 (0.93)	21.6 (0.95)	22.0 (1.25)
4	68.7 (1.73)	86.0 (2.18)	26.8 (1.03)	25.8 (1.39)
5	71.4 (4.80)	90.9 (3.58)	15.8 (0.40)	17.9 (0.73)
6	78.6 (1.89)	93.3 (1.88)	16.7 (2.49)	13.4 (1.86)
7	90.3 (1.59)	113.3 (4.12)	25.8 (1.81)	27.9 (0.62)
8	79.8 (0.89)	96.8 (0.43)	22.1 (0.15)	22.6 (0.47)
9	75.2 (0.72)	99.0 (2.04)	15.3 (0.61)	23.8 (1.42)
10	74.8 (3.98)	98.4 (7.86)	19.7 (0.65)	20.2 (2.03)
11	72.0 (3.21)	85.2 (4.88)	13.4 (0.64)	14.7 (1.58)
12	82.4 (1.63)	105.8 (4.87)	16.4 (1.79)	14.0 (0.90)
13	82.5 (2.94)	104.6 (2.58)	20.4 (0.96)	25.0 (0.42)
14	86.9 (1.97)	113.6 (5.35)	/	24.0 (1.15)
15	75.3 (2.80)	96.4 (1.04)	17.9 (2.22)	19.9 (0.74)
16	70.8 (5.54)	101.5 (1.68)	15.3 (0.63)	17.1 (1.66)

**Table 12:** Range analysis of mechanical properties and SD results (MPa).

Index	Level	W/B (SD)	CA (SD)	SF (SD)	CCW (SD)
Compressive strength	K1	90.8 (3.31)	96.8 (5.28)	93.1 (5.87)	106.4 (8.58)
	K2	98.6 (5.79)	99.2 (9.75)	96.2 (6.23)	94.5 (5.64)
	K3	97.1 (10.65)	96.9 (6.54)	100.5 (5.82)	95.9 (8.85)
	K4	104.0 (6.26)	97.5 (5.61)	100.6 (9.49)	93.7 (8.43)
Flexural strength	K1	19.4 (2.64)	20.0 (2.49)	14.6 (3.49)	19.8 (2.44)
	K2	20.4 (2.12)	18.5 (2.98)	17.1 (1.38)	19.7 (1.71)
	K3	18.2 (3.08)	21.1 (2.24)	23.1 (2.27)	19.3 (3.00)
	K4	21.5 (2.19)	19.9 (2.39)	24.7 (2.57)	20.7 (3.17)

**Table 13:** ANOVA results of mechanical properties.

Index	Factor	SS	DOF	MS	F	Fa(3,3)	Significance
Compressive strength	W/B	355.99	3	118.66	2.77	0.01 29.46	-
	CA	15.33	3	5.11	0.12	0.05 9.28	-
	SF	156.83	3	52.28	1.22	0.1 5.39	-
	CCW	418.46	3	139.49	3.26	-	-
	Error	128.40	3	42.80	-	-	-
Flexural strength	W/B	24.28	3	8.09	1.24	0.01 29.46	-
	CA	13.85	3	4.62	0.71	0.05 9.28	-
	SF	277.17	3	92.39	14.13	0.1 5.39	(*) *
	CCW	4.36	3	1.45	0.22	-	-
	Error	19.62	3	6.54	-	-	-

Note: (*) indicates significance at the 90% confidence level; * indicates significance at the 95% confidence level.

**Table 14:** Range analysis of fiber dispersion coefficient and SD results.

Level	W/B (SD)	CA (SD)	SF (SD)	CCW (SD)
K1	0.732 (0.102)	0.703 (0.096)	0.691 (0.118)	0.697 (0.118)
K2	0.729 (0.099)	0.710 (0.120)	0.698 (0.119)	0.708 (0.104)
K3	0.673 (0.144)	0.688 (0.072)	0.733 (0.089)	0.707 (0.030)
K4	0.699 (0.084)	0.731 (0.138)	0.711 (0.109)	0.721 (0.131)

**Table 15:** Tensile strength and SD results (MPa).

Specimen Groups	Tensile Strength (SD)
SF0CCW0	4.7 (0.31)
SF0CCW1	5.3 (0.22)
SF0CCW2	4.8 (0.09)
SF1CCW0	4.8 (0.10)
SF1CCW1	5.3 (0.12)
SF1CCW2	4.1 (0.28)
SF2CCW0	7.2 (0.40)
SF2CCW1	7.4 (0.18)
SF2CCW2	6.2 (0.10)

## Data Availability

The original contributions presented in this study are included in the article. Further inquiries can be directed to the corresponding author.
